# OsALKBH9‐mediated m^6^A demethylation regulates tapetal PCD and pollen exine accumulation in rice

**DOI:** 10.1111/pbi.14354

**Published:** 2024-04-17

**Authors:** Jun Tang, Dekun Lei, Junbo Yang, Shuyan Chen, Xueping Wang, Xiaoxin Huang, Shasha Zhang, Zhihe Cai, Shanshan Zhu, Jianmin Wan, Guifang Jia

**Affiliations:** ^1^ Synthetic and Functional Biomolecules Center, Beijing National Laboratory for Molecular Sciences, Key Laboratory of Bioorganic Chemistry and Molecular Engineering of Ministry of Education, College of Chemistry and Molecular Engineering Peking University Beijing China; ^2^ Institute of Animal Sciences Chinese Academy of Agricultural Sciences Beijing China; ^3^ National Key Facility for Crop Gene Resources and Genetic Improvement Institute of Crop Sciences, Chinese Academy of Agricultural Sciences Beijing China; ^4^ Shenzhen Branch, Guangdong Laboratory of Lingnan Modern Agriculture, Genome Analysis Laboratory of the Ministry of Agriculture and Rural Affairs Agricultural Genomics Institute at Shenzhen, Chinese Academy of Agricultural Sciences Shenzhen Guangdong China; ^5^ Peking‐Tsinghua Center for Life Sciences, Peking University Beijing China; ^6^ Beijing Advanced Center of RNA Biology Peking University Beijing China

**Keywords:** m^6^A, demethylase, tapetal PCD, exine, OsALKBH9, rice

## Abstract

The *N* ^6^‐methyladenosine (m^6^A) mRNA modification is crucial for plant development and stress responses. In rice, the male sterility resulting from the deficiency of OsFIP37, a core component of m^6^A methyltransferase complex, emphasizes the significant role of m^6^A in male fertility. m^6^A is reversible and can be removed by m^6^A demethylases. However, whether mRNA m^6^A demethylase regulates male fertility in rice has remained unknown. Here, we identify the mRNA m^6^A demethylase OsALKBH9 and demonstrate its involvement in male fertility regulation. Knockout of *OsALKBH9* causes male sterility, dependent on its m^6^A demethylation activity. Cytological analysis reveals defective tapetal programmed cell death (PCD) and excessive accumulation of microspores exine in *Osalkbh9‐1*. Transcriptome analysis of anthers shows up‐regulation of genes involved in tapetum development, sporopollenin synthesis, and transport pathways in *Osalkbh9‐1*. Additionally, we demonstrate that OsALKBH9 demethylates the m^6^A modification in *TDR* and *GAMYB* transcripts, which affects the stability of these mRNAs and ultimately leads to excessive accumulation of pollen exine. Our findings highlight the precise control of mRNA m^6^A modification and reveal the pivotal roles played by OsALKBH9‐mediated m^6^A demethylation in tapetal PCD and pollen exine accumulation in rice.

## Introduction

Rice (*Oryza sativa*) is a prominent crop globally and serves as a monocot model. The development of pollen in anthers is a highly regulated process, which involves the formation of male meiocytes, gametophytes and gametes (Zhang and Yang, 2014). Tapetum, the innermost layer of the anther wall, supports gametogenesis and undergoes programmed cell death (PCD) after meiosis (Zhang and Yang, 2014). Abnormal development and/or delayed or premature degradation of the tapetum cells results in male sterility. In rice, the development and PCD of tapetum cells are controlled by a highly conserved complex network of transcription factors (TFs): OsUDT1 (UNDEVELOPED TAPETUM 1), OsTDF1 (DEFECTIVE in TAPETAL DEVELOPMENT and FUNCTION1), OsTDR (TAPETUM DEGENERATION RETARDATION), OsMS188 (Male‐sterile 188) and OsPTC1 (PERSISTENT TAPETAL CELL1) (Yao *et al*., 2022). Any genetic mutations in these five genes can lead to male sterility and abnormalities in tapetal development and/or PCD. Additionally, OsEAT1 (ETERNAL TAPETUM 1), another bHLH TF, plays a crucial role in tapetal PCD by directly regulating the transcription of two aspartic protease encoding genes, *OsAP25* and *OsAP27* (Niu *et al*., 2013). Furthermore, OsGAMYB, a MYB TF regulated by gibberellin (GA), also contributes to tapetal development and PCD (Aya *et al*., 2009).

The pollen wall serves a critical function in safeguarding male gametes from multiple environmental stresses. It is composed of two layers, intine and exine, which consist primarily of polysaccharides and lipidic sporopollenin, respectively (Shi *et al*., 2015). Sporopollenin is synthesized within the tapetum cells and subsequently transported to the surface of microspores for exine formation (Shi *et al*., 2015). In Arabidopsis, sporopollenin biosynthesis and exine accumulation are regulated by a series of TFs which form a regulatory cascade (AtDYT1‐AtTDF1‐AtAMS‐AtMS188‐AtMS1) activating transcription of their downstream TFs and target genes (Yao *et al*., 2022). In rice, cascade (OsUDT1‐OsTDF1‐OsTDR‐OsMS188‐OsPTC1) also regulates exine formation (Yao *et al*., 2022). OsMS188 and OsGAMYB operate several genes involved in sporopollenin synthesis or transport, enabling them to directly activate gene expression, such as *PTC1*, *CYP703A3*, *CYP704B2*, *ABCG15* and *PKS1* (Aya *et al*., 2009; Jin *et al*., 2022).

*N* ^6^‐methyladenosine (m^6^A) is the most prevalent chemical modification in mRNA, and it regulates gene expression at both transcriptional and post‐transcriptional levels (Tang *et al*., 2023). m^6^A is installed, removed and recognized by methyltransferases (writers), demethylases (erasers) and m^6^A‐binding proteins (readers), respectively (Tang *et al*., 2023). In rice, male fertility is significantly impacted by mRNA m^6^A writers (Cheng *et al*., 2022; Zhang *et al*., 2019). Knocking out OsFIP37, a core component of m^6^A methyltransferase complex, leads to abnormal meiosis and early degeneration of microspores during the vacuolated pollen stage (Cheng *et al*., 2022; Zhang *et al*., 2019). Moreover, the absence of OsEDM2L (ENHANCED DOWNY MILDEW 2‐LIKE), a protein containing an *N* ^6^‐adenine methyltransferase‐like domain vital for proper mRNA m^6^A modification in anthers, causes defective pollen development and delayed tapetal PCD (Ma *et al*., 2021). m^6^A is a reversible mRNA modification that can be demethylated by m^6^A demethylases. Several m^6^A demethylases have been identified in Arabidopsis and tomato, including ALKBH10B (Duan *et al*., 2017), ALKBH9B (Martinez‐Perez *et al*., 2017) and SlALKBH2 (Zhou *et al*., 2019). These demethylases are involved in various biological processes such as floral transition (Duan *et al*., 2017), abiotic stress response (ALKBH10B) (Shoaib *et al*., 2021; Tang *et al*., 2021), alfalfa mosaic virus (AMV) infection (Martinez‐Perez *et al*., 2017) and abscisic acid (ABA) response (ALKBH9B) (Tang *et al*., 2022), and tomato fruit ripening (SlALKBH2) (Zhou *et al*., 2019). However, the biological functions of demethylase in rice development remain unknown, despite its crucial role.

Here, we identified OsALKBH9 as a rice mRNA m^6^A demethylase. Loss of function of OsALKBH9 results in male sterility, delayed tapetal degradation, and excessive accumulation of pollen exine. OsALKBH9 is highly expressed in anthers and regulates the expression of genes involved in tapetal development and pollen exine accumulation. Our findings demonstrate that OsALKBH9 mediates the m^6^A demethylation of *TDR* and *GAMYB* mRNAs, leading to decreased stability and repressing of downstream gene expression, such as *OsMS188*, *PTC1*, *CYP703A3*, *CYP704A4*, *PKS2* and *ABCG15*. These insights provide valuable information on the vital roles of m^6^A in controlling rice fertility and offer male sterile materials that can aid in hybrid breeding.

## Results

### Knock out of *OsALKBH9* caused male sterility in rice

**Figure 1:**
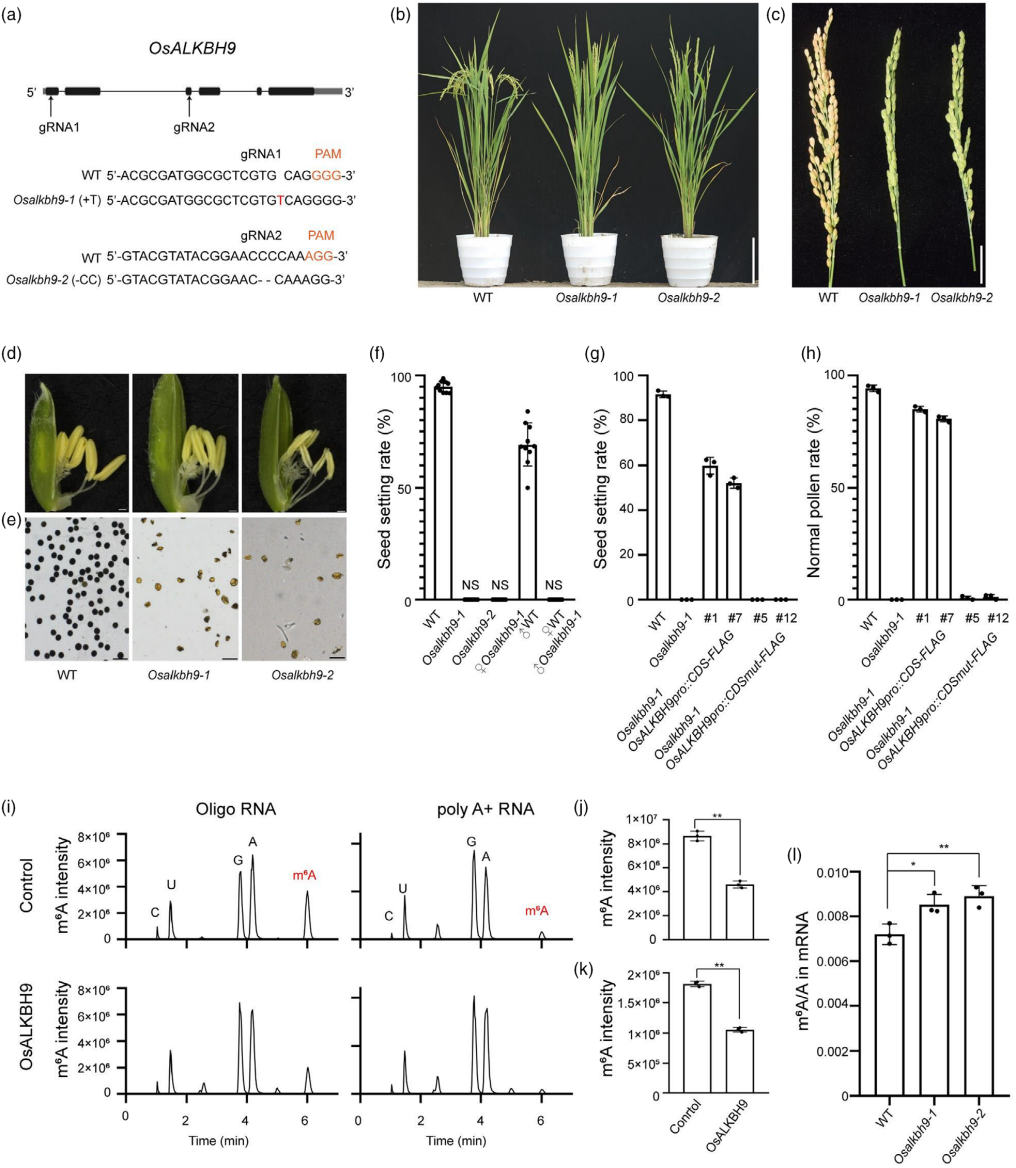
The regulation of OsALKBH9 in male fertility depends on its m^6^A demethylase activity. (a) CRISPR/Cas9‐mediated target mutagenesis of *OsALKBH9*. The upper panel shows the *OsALKBH9* genomic region and the two CRISPR/Cas9 target sites indicated by arrows. Exons and other sequences are indicated by black boxes and lines, respectively. The lower panel shows alignment of wild‐type (WT), *Osalkbh9‐1* and *Osalkbh9‐2* sequences containing the CRISPR/Cas9 target sites. *Osalkbh9‐1* and *Osalkbh9‐2* contain a 1‐bp insertion of T (red) and a 2‐bp deletion of C (dash), respectively. (b) The phenotype of WT, *Osalkbh9‐1* and *Osalkbh9‐2* mutant plants after heading. Scale bar, 20 cm. (c) Comparison of WT, *Osalkbh9‐1* and *Osalkbh9‐2* panicles at the mature stage. Scale bar, 2 cm. (d) Spikelets of WT, *Osalkbh9‐1* and *Osalkbh9‐2* plants. Scale bars, 0.5 mm. (e) Pollen grains of WT, *Osalkbh9‐1* and *Osalkbh9‐2* stained by I_2_/KI solution. Scale bars, 50 μm. (f) The seed setting rates of the mutants crossed with WT plants (*n* = 10 plants). (g‐h) The seed setting rates (g) and pollen grains I_2_/KI solution (h) of genetically complementary plants and demethylase inactive complementary plants (*n* = 3 plants). The results of two representative lines of genetically complementary plants and demethylase inactive complementary plants were present, individually. (i–k) Recombinant OsALKBH9 protein demethylates the m^6^A modification in m^6^A‐containing ssRNA and poly A^+^ RNA extracted from rice *in vitro*. *n* = 3 biological replicates. (i) LC–MS/MS chromatograms of digested substrates of *in vitro* demethylase activity reaction. (j, k) m^6^A changes using ssRNA (j) and poly A^+^ RNA (k) as substrates after *in vitro* demethylase activity reaction. (l) m^6^A percentage relative to adenosine (m^6^A/A ratio) determined by LC–MS/MS in mRNA purified from 14‐day‐old shoots of WT, *Osalkbh9‐1* and *Osalkbh9‐2*. *n* = 3 biological replicates. Data are means ± SD for three biological replicates. Student's *t* test: *(*P* < 0.05), **(*P* < 0.01).

To identify mRNA m^6^A demethylases and explore their functions in rice, we searched for homologues of mammalian m^6^A demethylase ALKBH5. We designated two proteins predicted as putative m^6^A demethylases (LOC_Os06g04660 and LOC_Os05g33310) as OsALKBH9 and OsALKBH10, respectively (Figure S1). Using clustered regularly interspaced short palindromic repeats (CRISPR)/CRISPR‐associated nuclease 9 (Cas9) mediated gene editing in the Nipponbare cultivar background (Oryza sativa ssp. Japonica), we obtained two mutants of *OsALKBH9* with predicted early termination of translation, which were named *Osalkbh9‐1* and *Osalkbh9‐2* (Figure 1a). Although both mutants exhibited normal vegetative growth, they failed to produce seeds (Figure 1b, c, f). By CRISPR‐Cas9 technology, we also obtained two mutants of *OsALKBH10*, which caused predicted early termination of translation (Figure S2). However, we did not find obvious development phenotypes in the *Osalkbh10* mutants. So, we focused on the research of OsALKBH9. Reciprocal cross‐pollination between *Osalkbh9‐1* and the wild‐type (WT) plants showed that all WT ovules were unable to be fertilized by *Osalkbh9‐1* pollen grains, whereas the ovules of *Osalkbh9‐1* were fertilized by WT pollen grains (Figure 1f), indicating that the lack of seeds in *Osalkbh9* mutant is due to the defective pollen grains. Compared to the WT, the anthers of both mutants did not fill up and became pale (Figure 1d), and the pollen grains were shrunken and lacked starch inclusions (Figure 1e), indicating male sterile of *Osalkbh9* mutants. To test the genetic action of the *Osalkbh9* mutation, we calculated the ratio of sterile plants to fertile plants and determined the genotype of the inbred offspring of heterozygous mutants (CRISPR‐Cas9 T‐DNA free). The three genotypes, *OsALKBH9*/*OsALKBH9* (WT): *OsALKBH9*/*Osalkbh9‐1* (heterozygote) and *Osalkbh9‐1*/*Osalkbh9‐1* (homozygote), segregated at a ratio of 1:2:1, and the phenotypic ratio of male fertility to male sterility fit a 3:1 ratio, with co‐segregation between the genotypes and phenotypes (Table S1). These results indicate that the *Osalkbh9* mutation acted in a recessive sporophytic manner.

We conducted genetic complementation experiments to further confirm the function of OsALKBH9 in pollen development. We transformed *Osalkbh9‐1*/*Osalkbh9‐1* calli with the construct of *OsALKBH9pro*::*OsALKBH9*CDS‐FLAG, which contains a 2.1 kb native promoter and a 1.9 kb coding sequence fused in‐frame with a 3 × FLAG tag. In the T_0_ generation of complementation lines, the seed setting rate and pollen activity were largely restored (Figure 1g, h; Figure S3), confirming the male‐sterile phenotype of *Osalkbh9* mutants is caused by the loss of function of OsALKBH9.

### The regulation of OsALKBH9 in male fertility depends on its m^6^A demethylase activity

To verify if OsALKBH9 functions as an mRNA m^6^A demethylase, we expressed full‐length OsALKBH9 with a His‐tag in *Escherichia coli* as fusion proteins. Demethylation assays were performed with the purified proteins by incubating overnight with synthetic 14‐mer m^6^A‐modified RNA or the full‐length mRNA isolated from rice seedlings. The nucleosides that were digested from the reaction products were analysed by Liquid chromatography–tandem mass spectrometry (LC–MS/MS). The results indicated that approximately 50% of the m^6^A present in either synthetic ssRNA or rice mRNA was removed by OsALKBH9 *in vitro* (Figure 1i–k), clearly demonstrating its RNA m^6^A demethylation activity *in vitro*. Furthermore, we observed an increase in the level of mRNA m^6^A modification in 14‐day‐old shoots of both *Osalkbh9‐1* and *Osalkbh9‐2* compared to WT (Figure 1l), verifying the *in vivo* m^6^A demethylation activity of OsALKBH9 in rice.

To determine whether the regulation of male fertility by OsALKBH9 depends on its putative RNA demethylase activity, we conducted complementation experiments using the *OsALKBH9pro*::*OsALKBH9CDSmut‐FLAG* (H324A/D326A) construct which consists of the catalytically inactive OsALKBH9 coding sequence (Duan *et al*., 2017). We obtained 20 independent transgenic lines, but none of them showed any restoration of the seed‐setting rate or pollen fertility (Figure 1g, h). These results suggest that the male sterile phenotype of *Osalkbh9* mutant is directly linked to the RNA demethylase activity of OsALKBH9 being compromised.

### OsALKBH9 is required for the tapetal PCD and pollen exine accumulation

**Figure 2:**
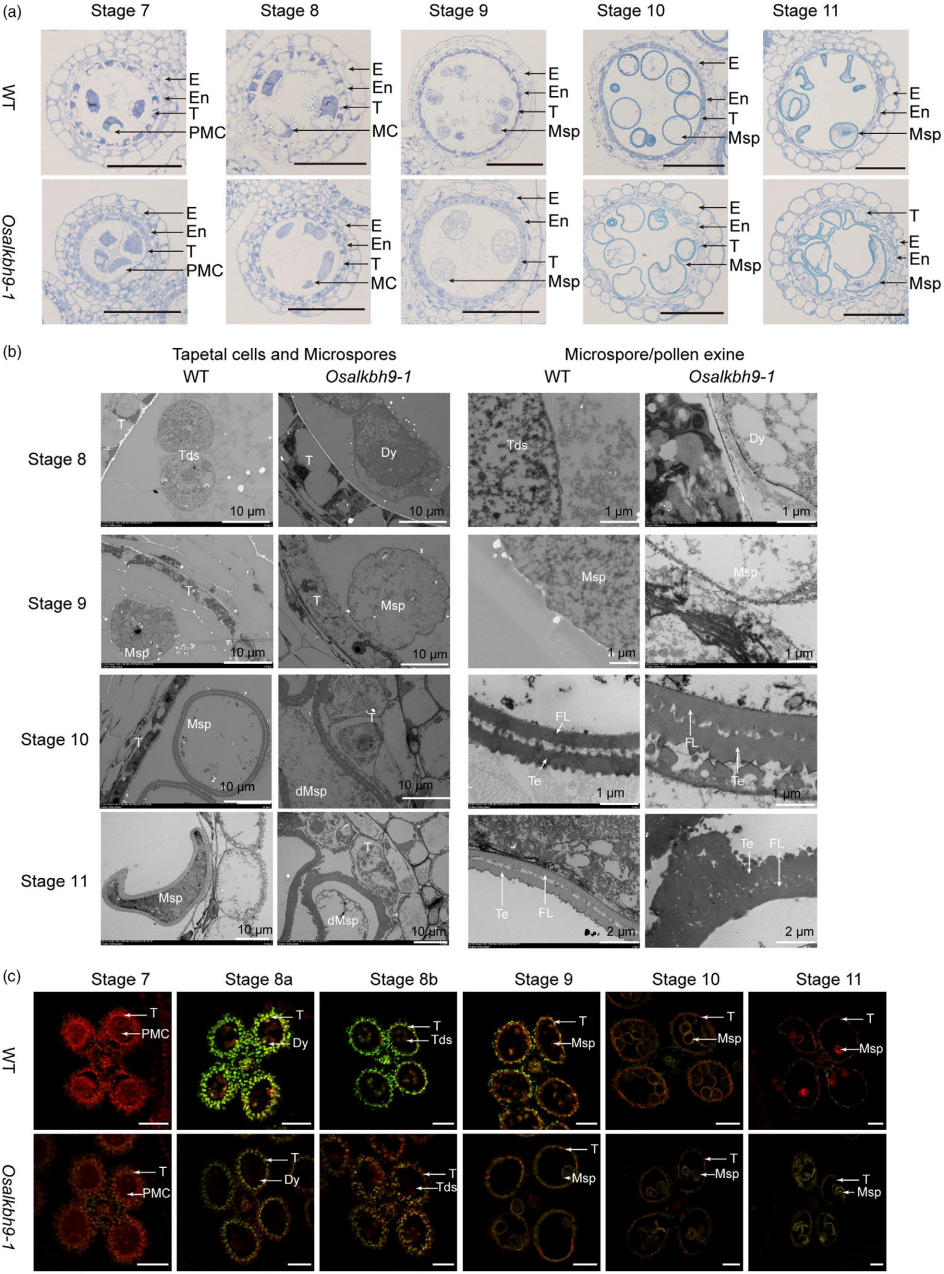
Knockout of *OsALKBH9* caused delayed degradation of tapetal cells and abnormal pollen wall patterning. (a) Semi‐thin sections of anthers at stage 7 to stage 11 in the wild type and *Osalkbh9‐1* mutant. The images show a single locule of an anther. Scale bars, 50 μm. (b) Transmission electron micrographs of the wild‐type and *Osalkbh9‐1* anthers from stage 8 to stage 11. (c) TUNEL analyses of WT and *Osalkbh9‐1* anthers at stage 7 to stage 11. Propidium iodide staining is indicated by red fluorescence and TUNEL‐positive staining is indicated by yellow to green fluorescence. Scale bars, 50 μm. E, epidermis; En, endothecium; T, tapetum; PMC, pollen mother cell; MC, meiotic cell; Msp, microspore; dMsp, aborted microspore; Tds, tetrads; Dy, dyad cell; FL, foot layer; Te, tectum.

To investigate the cellular abnormality of *Osalkbh9‐1* during male reproductive development, anther transverse sections were examined at different developmental stages. From stage 7 to stage 8 (as labelled by (Zhang *et al*., 2011)), no obvious differences were observed between anthers from WT and *Osalkbh9‐1* (Figure 2a). At stage 9, the tapetum of WT was condensed, forming a thin cell layer, while that of *Osalkbh9‐1* remained large (Figure 2a). At stage 10, the tapetum in WT became more concentrated, degraded and thinned, and the microspores underwent vacuolation (Figure 2a). Conversely, in *Osalkbh9‐1*, the degradation of tapetal cells was abnormal, with some cells remaining undegraded, and the microspores appeared irregularly shaped (Figure 2a). At stage 11, only a small amount of degradation residue was left in the tapetum of WT anthers, and the microspore vacuoles enlarged (Figure 2a). However, there were still undegraded tapetal cells, and all the microspores were aborted in *Osalkbh9‐1* (Figure 2a).

Transmission electron microscopy (TEM) was utilized to investigate the developmental defects in the tapetum and microspores of *Osalkbh9‐1*. While the tapetum of WT continued to degrade until complete degradation from stage 9 to stage 11, intact tapetal cells were still evident in *Osalkbh9‐1* at stage 11, which corroborates with the transverse sections (Figure 2b). From stage 10 to stage 11, both *Osalkbh9‐1* and WT microspores were enclosed by a double‐layered exine consisting of tectum and nexine (Figure 2b); however, the exine of *Osalkbh9‐1* was thicker than that of WT (Figure 2b). At stage 10, the exine structure of *Osalkbh9‐1* resembled that of WT (Figure 2b); but the exine pattern of *Osalkbh9‐1* at stage 11 became irregular due to abnormal tectum accumulation (Figure 2b). These results indicate that OsALKBH9 is necessary for both pollen exine accumulation and appropriate patterning.

To investigate the disturbed PCD in *Osalkbh9‐1* anthers, the terminal deoxynucleotidyl transferase‐mediated dUTP‐biotin nick end labelling (TUNEL) assay was performed due to the delayed tapetum degradation in *Osalkbh9‐1*. At stage 7, no TUNEL signals were observed in the anthers of either WT or *Osalkbh9‐1* mutant (Figure 2c). However, strong TUNEL signals were observed in the tapetum of the WT at stage 8a and continued until stage 9, while TUNEL signals in *Osalkbh9‐1* were detected at stage 8a and stage 8b, but were weaker than those of the WT (Figure 2c), indicating inadequate degradation of tapetum cells in *Osalkbh9‐1* (Figure 2c). Additionally, the microspores from stage 9 to stage 11 in *Osalkbh9‐1* mutant but not in WT displayed TUNEL signals (Figure 2c). These results suggest abnormal tapetal PCD and microspores development in *Osalkbh9‐1*, further confirming the critical roles of OsALKBH9 in tapetal PCD, microspores development and microspores/pollen exine accumulation and patterning.

### OsALKBH9 is highly expressed in anthers and mainly localized in cytoplasm

**Figure 3:**
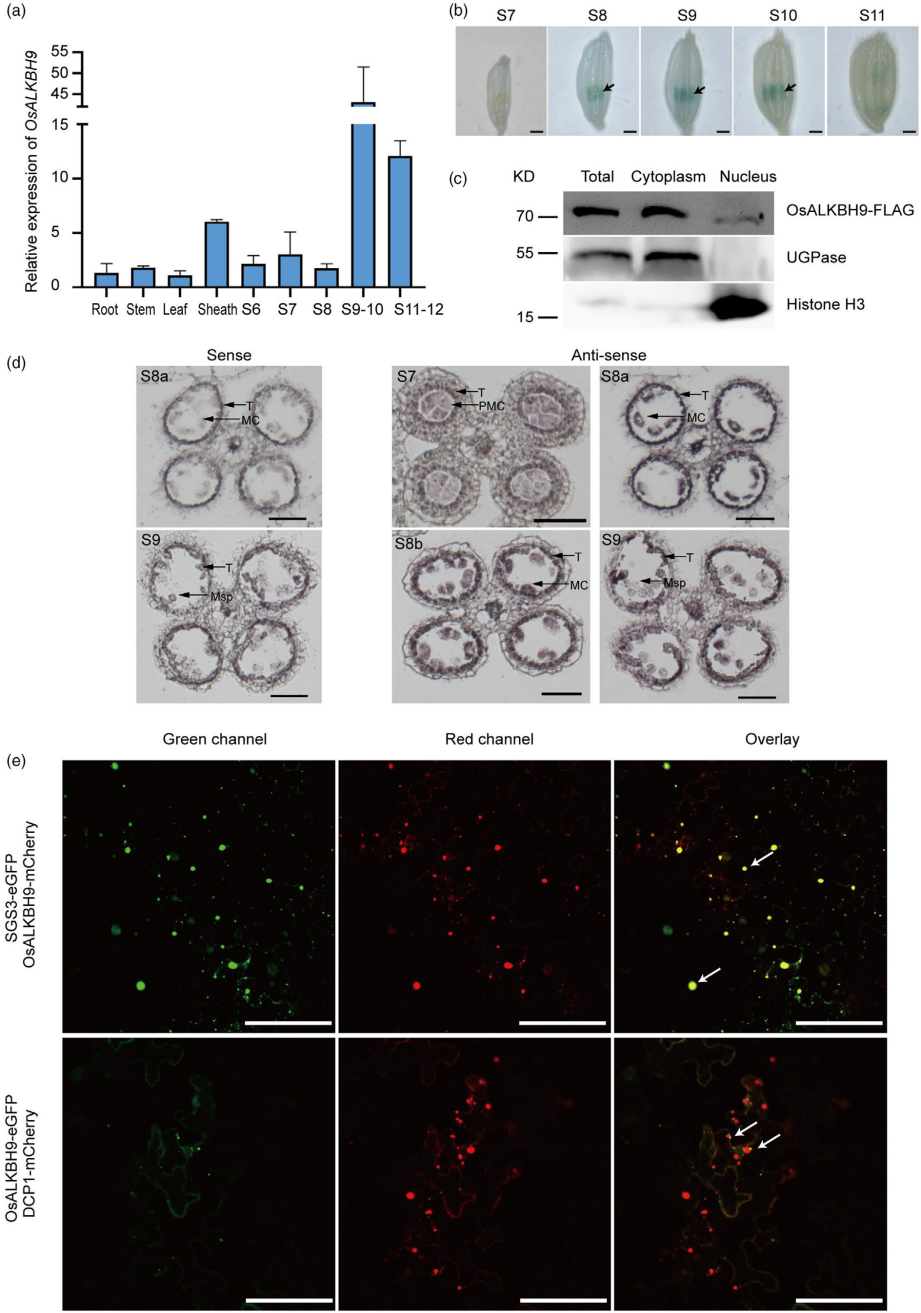
*OsALKBH9* is highly expressed in anthers and OsALKBH9 is mainly localized in cytoplasm. (a) Expression analysis of *OsALKBH9* in different tissues by qRT‐PCR. Anthers were collected at developmental stage 6 to stage 12. Other tissues were harvested from plants at the flowering stage. S6, stage 6; S7, stage 7; S8, stage 8; S9–10, stage 9 to stage 10; S11–12, stage 11 to stage 12. (b) GUS staining of transgenic anthers containing *OsALKBH9pro*::*GUS*. Scale bars, 1 mm. Arrows pointing anthers with strong GUS signals. S7, stage 7; S8, stage 8; S9, stage 9; S10, stage 10; S11, stage 11. (c) Subcellular fraction and immunoblot assay. Total protein, cytoplasm, and nuclei‐enriched fractions from *Osalkbh9‐1 OsALKBH9pro*::*OsALKBH9CDS‐FLAG* transformants are subject to SDS‐PAGE. UGPase and Histone H3 are used as cytoplasmic and nuclear markers, respectively. (d) *In situ* hybridization of *OsALKBH9* transcripts in WT anthers. S7, stage 7; S8a, stage 8a; S8b, stage 8b; S9, stage 9. T, tapetum; PMC, pollen mother cell; MC, meiotic cell; Msp, microspore. Scale bars, 50 μm. (e) Subcellular localization of OsALKBH9 in *N. benthamiana* leaves epidermal cells. Arrows pointing co‐localized foci of OsALKBH9‐mCherry and SGS3‐eGFP (the upper panel), and non‐co‐localized foci of OsALKBH9‐eGFP and DCP1‐mCherry (the lower panel). Scale bars, 100 μm.

To investigate the expression pattern of *OsALKBH9*, we measured its transcript levels in different tissues by qRT‐PCR. The results showed that *OsALKBH9* was highly expressed in anthers at stage 9 to stage 10 and stage 11 to stage 12 (Figure 3a). We further investigated the expression pattern of *OsALKBH9* during anthers development using *OsALKBH9pro*::*GUS* (β‐glucuronidase) transgenic plants. GUS staining revealed that *OsALKBH9* was highly expressed in anthers from stage 8 to stage 10 (Figure 3b). We also examined the *OsALKBH9* expression pattern by RNA *in situ* hybridization. The results showed that *OsALKBH9* transcripts are highly expressed in tapetal cells (S8a, S8b and S9), meiotic cells (S8a and S8b) and microspore (S9) (Figure 3d).

To determine the subcellular localization of OsALKBH9, we transiently expressed OsALKBH9‐GFP via agroinfiltration in *N. benthamiana* leaves. We found that OsALKBH9‐GFP exhibited a dotted cytoplasm distribution pattern (Figure S4), similar to the localization pattern of its homologues (AtALKBH9B) in *Arabidopsis thaliana* (Martinez‐Perez *et al*., 2017). We further confirmed the cytoplasmic localization of OsALKBH9 by isolating the cytoplasmic and nuclear components of *Osalkbh9‐1 OsALKBH9pro*::*OsALKBH9CDS‐FLAG* and determining its location through western blotting. The outcome demonstrated that almost all OsALKBH9 proteins are localized in the cytoplasm (Figure 3c). Because AtALKBH9B was founded co‐localized with DCP1 and SGS3 (Martinez‐Perez *et al*., 2017), which are marker proteins of P‐bodies and siRNA‐bodies. So, we checked whether OsALKBH9 co‐localized with DCP1 and SGS3, and the results showed that OsALKBH9 co‐localized with SGS3 but not with DCP1 (Figure 3e). Since the siRNA bodies commonly co‐localized with stress granules (SGs) (Jouannet *et al*., 2012; Kakutani *et al*., 2012), it suggests that the function of OsALKBH9 might involve in siRNA‐bodies and/or stress granules.

### *OsALKBH9* deficiency affects the expression of genes related to male reproductive development and exine accumulation

**Figure 4:**
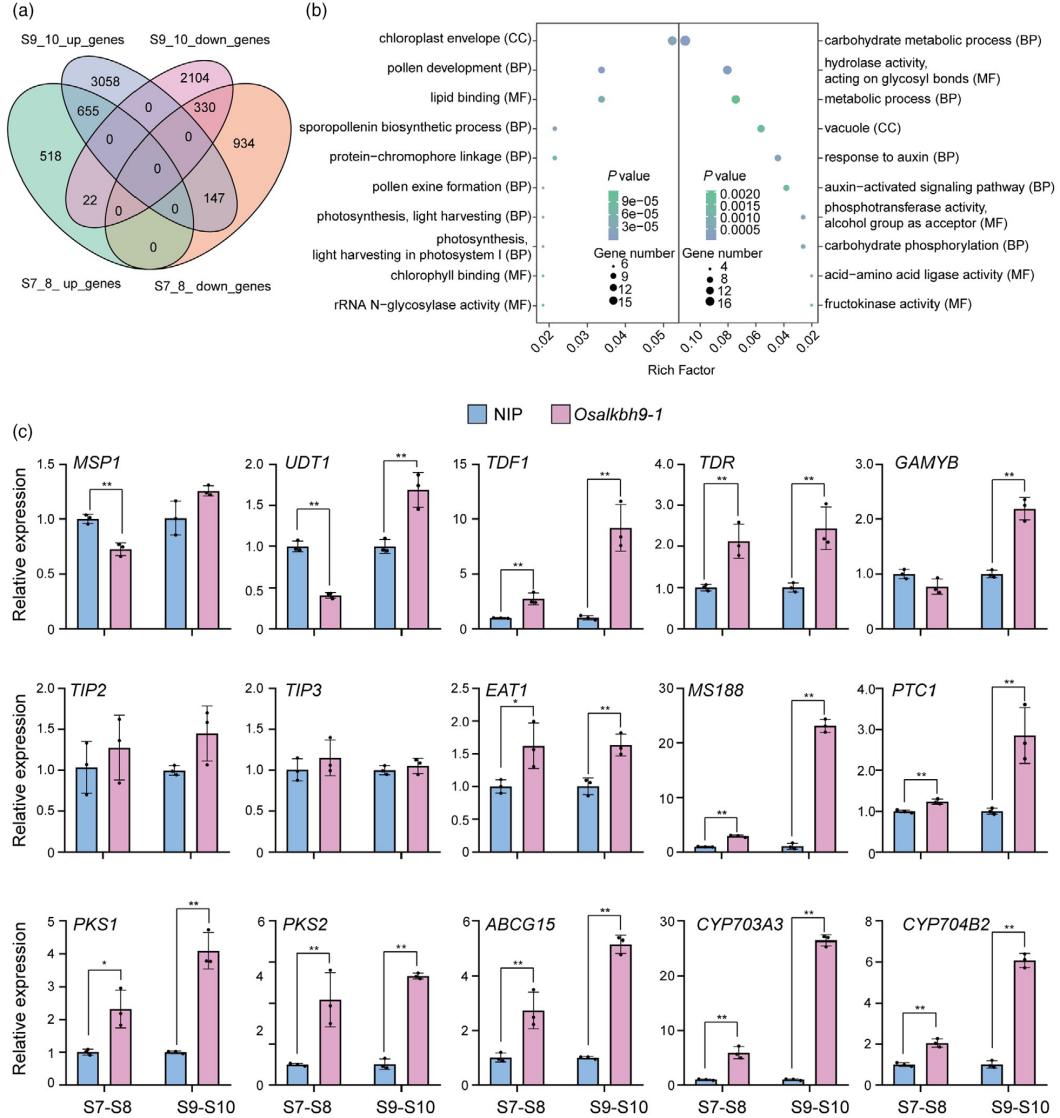
OsALKBH9 affects the expression of genes related to male reproductive development and exine accumulation. (a) Venn diagram depicting the differentially expressed genes (DEGs, *Osalkbh9‐1*/WT) at stage 7 to stage 8 and stage 9 to stage 10. (b) Gene ontology analysis of the co‐upregulated and co‐downregulated genes at stage 7 to stage 8 and stage 9 to stage 10. Left part indicates the co‐upregulated genes, while right part indicates the co‐downregulated genes. CC, cell component; BP, biological process; MF, molecular function. (c) qRT‐PCR detection of genes required for tapetum development and pollen wall accumulation. *UBQ* was used as an internal control. Data are means ± SD for three biological replicates. Student's *t* test: *(*P* < 0.05), **(*P* < 0.01).

As OsALKBH9 is highly expressed in anthers during stage 8 to stage 10, we conducted RNA‐seq analysis to evaluate its effect on male reproductive development. We compared gene expression profiles of stage 7 to stage 8 (Table S3) and stage 9 to stage 10 (Table S4) anthers between *Osalkbh9‐1* and the WT. Clustering analysis confirmed the robustness of our RNA‐seq data (Figures S5 and S6). Our analysis revealed differential expression of 2606 genes in stage 7 to stage 8 anthers (1195 upregulated, 1411 downregulated) and 6316 genes in stage 9 to stage 10 anthers (3860 upregulated, 2456 downregulated) (with the threshold criteria | Log_2_[fold‐change] | ≥0.585 and FDR <0.05) (Figure S7a,c). We performed Gene Ontology (GO) analysis and found that the upregulated genes in stage 7 to stage 8 anthers were primarily associated with lipid metabolism, fatty acid biosynthesis, pollen and sporopollenin development, and photosynthesis (Figure S7b), while those in stage 9 to stage 10 anthers were linked to photosynthesis, flower development, sporopollenin biosynthesis and pollen exine formation (Figure S7d). Downregulated genes were mainly involved in ribosome, translation, symporter activity and auxin response in stage 7 to stage 8 anthers (Figure S7b), whereas DNA replication and cell cycle were affected in stage 9 to stage 10 anthers (Figure S7d). We compared co‐upregulated and co‐downregulated genes in stages 7 to stage 8 and stage 9 to stage 10, revealing 985 co‐differentially expressed genes (655 upregulated and 330 downregulated) (Figure 4a). GO analysis showed that the upregulated genes were primarily associated with sporopollenin biosynthesis, pollen development and pollen exine formation, while the downregulated genes were linked to carbohydrate metabolic and auxin response (Figure 4b). We further utilized qRT‐PCR to detect gene expression related to tapetum development and sporopollenin synthesis/transport pathways (Figure 4c; Table S2). The results demonstrated upregulation of TFs *TDF1*, *TDR*, *EAT1*, *MS188* and *PTC1* at both stage 7 to stage 8 and stage 9 to stage 10 and *GAMYB* at stage 9 to stage 10 in *Osalkbh9‐1* (Figure 4c). Furthermore, the sporopollenin synthesis and transport genes *CYP703A3*, *CYP704A4*, *PKS2* and *ABCG15* were also upregulated at both stages (Figure 4c). These results suggest that the increased expression of genes involved in microspores sporopollenin accumulation in *Osalkbh9‐1* could account for the excessive accumulation of pollen exine in the mutant.

### Disruption of *OsALKBH9* leads to transcriptome‐wide m^6^A hypermethylation

**Figure 5:**
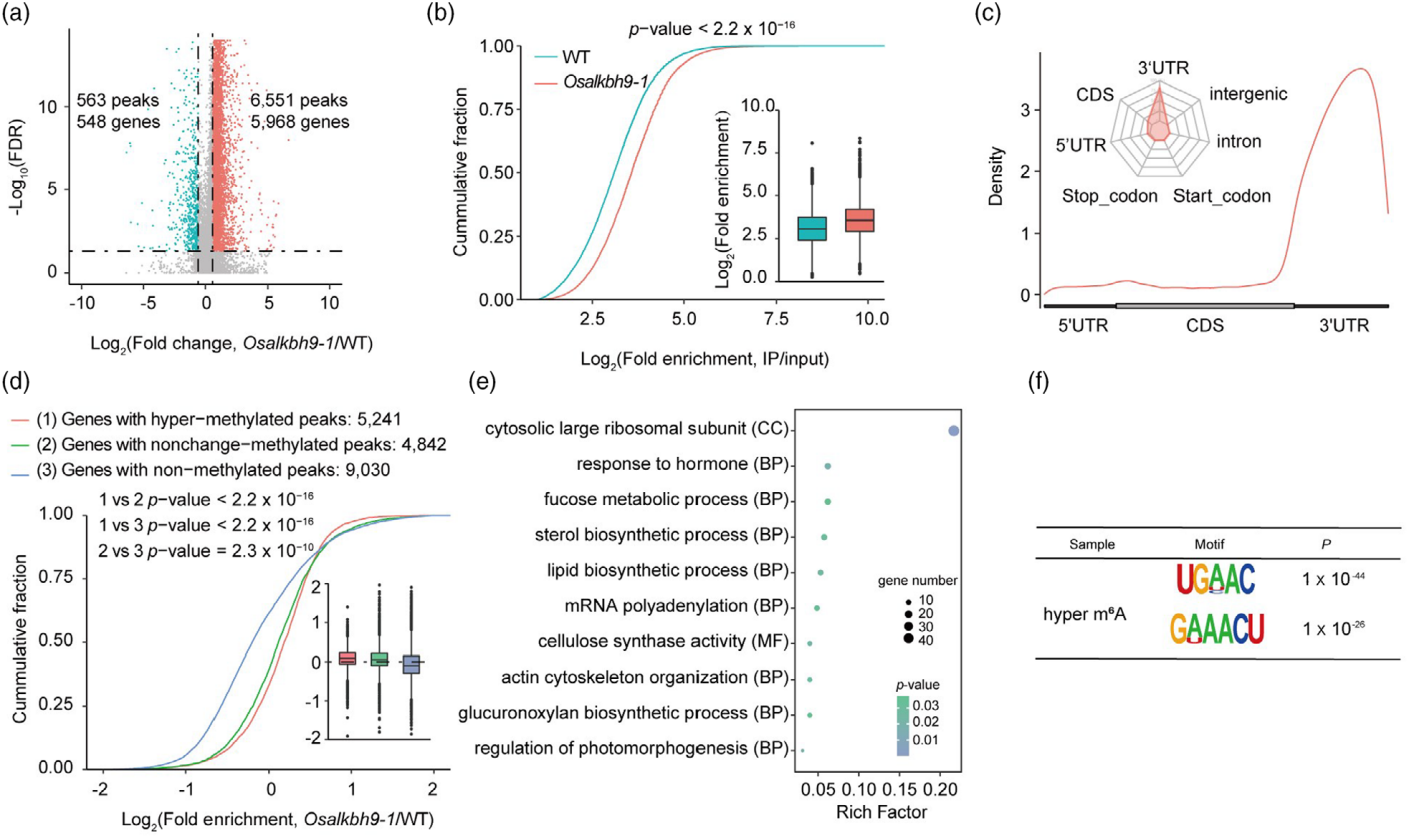
Loss of function of OsALKBH9 caused transcriptome‐wide m^6^A hypermethylation. (a) The result of the volcano plot shows the changes in m^6^A modification between *Osalkbh9‐1* and WT. (b) The results of cumulative fraction and boxplot show the log_2_(fold enrichment) of m^6^A peaks in *Osalkbh9‐1* and WT. The Kolmogorov–Smirnov test revealed a *P*‐value of less than 2.2 × 10^−16^. (c) The results of metaplot and radar plot represent the distribution of identified m^6^A hyper‐methylated peaks in *Osalkbh9‐1* across the indicated mRNA segments. (d) The cumulative fraction and boxplots illustrate the gene expression patterns of hyper‐methylated, non‐change methylated and hypo‐methylated peaks in *Osalkbh9‐1*/WT The Kolmogorov–Smirnov test revealed a *P*‐value of less than 2.2 × 10^−16^. (e) Gene ontology analysis was performed on the genes with hyper‐methylated peaks. (f) OsALKBH9‐dependent motifs were identified by using HOMER based on the top 1000 peaks.

We conducted m^6^A‐seq analysis on anthers at stage 9 to stage 10 from both *Osalkbh9‐1* and WT to investigate changes in m^6^A methylation patterns (Table S5). Our analysis revealed 6551 hyper‐methylated m^6^A peaks corresponding to 5968 genes and 563 hypo‐methylated peaks corresponding to 548 genes in the *Osalkbh9‐1* mutant (Figure 5a). Comparing m^6^A enrichment between WT and *Osalkbh9‐1*, we found significantly higher m^6^A enrichment in *Osalkbh9‐1* than in WT (Figure 5b), suggesting that disruption of OsALKBH9 leads to hyper‐methylation of m^6^A. Analysis of the metagene profiles revealed highly enriched hyper‐methylated m^6^A peaks in the 3′ untranslated region (3′ UTR) of transcripts (Figure 5c). Further analysis of hyper‐methylated m^6^A peaks within five non‐overlapping transcript regions showed that they were primarily enriched in the 3′ UTR (>75%) (Figure 5c). The enriched m^6^A motifs (UGHAC and GWAACU) observed in hyper‐methylated m^6^A peaks were consistent with the previously reported RRACH motif (Figure 5f).

We next investigated whether altered m^6^A levels resulting from *OsALKBH9* deficiency affect gene expression. Analysis of transcript accumulation in genes with non‐methylated, unchanged‐methylated and hyper‐methylated peaks indicated that genes with hyper‐methylated peaks were more likely to be upregulated than those without (Figure 5d). GO analysis revealed that hyper‐methylated genes were mainly associated with hormone response, regulation of photomorphogenesis, lipid biosynthetic and mRNA polyadenylation (Figure 5e). Joint analysis of m^6^A‐seq and RNA‐seq data identified 865 upregulated and 499 downregulated genes containing hyper‐methylated peaks, as well as 35 upregulated and 131 downregulated genes with hypo‐m^6^A methylation (Figure S8a). GO analysis demonstrated that hyper‐methylated and up‐regulated genes were enriched in pathways such as positive regulation of transcription, signal transduction and transcription coactivator activity, while hyper‐methylated and down‐regulated genes were enriched in carbohydrate metabolic processes and carbohydrate phosphorylation (Figure S8b). Overall, our study demonstrates that disruption of *OsALKBH9* results in transcriptome‐wide m^6^A hypermethylation.

### Loss of function of *OsALKBH9* caused m^6^A hypermethylation in *TDR* and *GAMYB* transcripts

**Figure 6:**
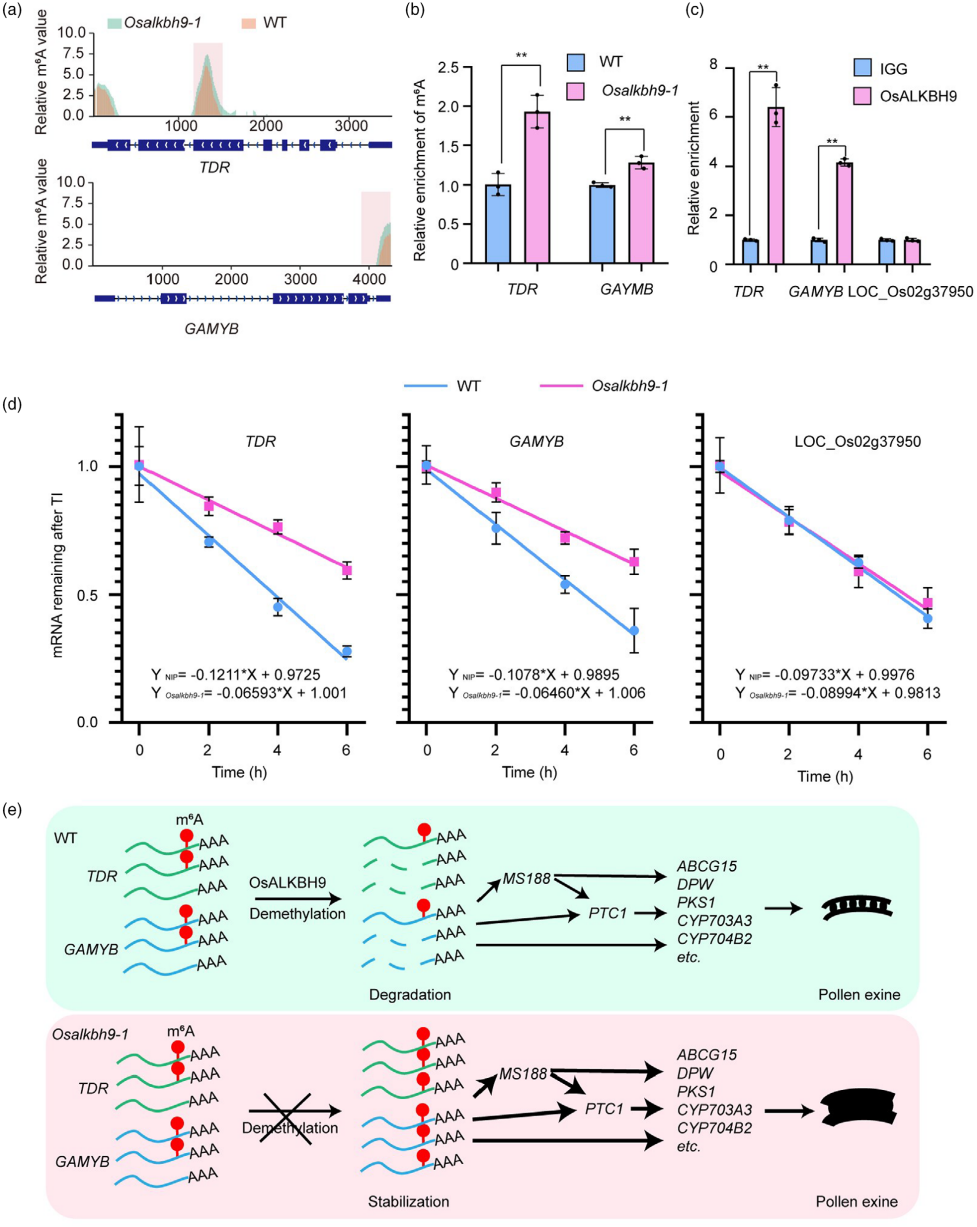
OsALKBH9‐dependent m^6^A demethylation regulates pollen exine accumulation. (a) The relative m^6^A value of *TDR* and *GAMYB*. The relative m^6^A value of *TDR* and *GAMYB* is determined using log_2_ (*Osalkbh9‐1*/NIP, reads count (IP/input)). (b) m^6^A‐IP‐qPCR results showing the relative m^6^A levels in stage 9 to stage 10 anthers of WT and *Osalkbh9‐1*. (c) RIP‐qPCR assays in *Osalkbh9‐1 OsALKBH9pro*::*OsALKBH9CDS‐FLAG* spikelets (anthers at stage 7 to stage 12) showing that OsALKBH9 directly binds to the *TDR* and *GAMYB* transcripts. (d) mRNA lifetimes of *TDR* and *GAMYB* in WT and *Osalkbh9‐1* spikelets (anthers at stage 7 to stage 12). LOC_Os02g37950 was used as the negative control. 18S RNA was used as a reference. TI, transcription inhibition. Data are means ± SD for three biological replicates. Student's *t* test: *(*P* < 0.05), **(*P* < 0.01). (e) A proposed working model for how OsALKBH9 modulates pollen exine accumulation. In wild type, OsALKBH9 removes m^6^A on *TDR* and *GAMYB* transcripts, reducing their mRNA stability and ensuring appropriate accumulation of pollen exine. In *Osalkbh9‐1*, *TDR* and *GAMYB* transcripts are m^6^A hypermethylated, promoting the stability of their mRNA, activating the expression of genes involved in pollen exine accumulation, leading to excessive accumulation of pollen exine and abnormal exine patterning.

We observed higher m^6^A peaks in the exon of *TDR* and 3′ UTR of *GAMYB* in *Osalkbh9‐1*, both of which are involved in tapetum and pollen exine development (Figure 6a). We also performed m^6^A‐immunoprecipitation followed by quantitative PCR (m^6^A‐IP‐qPCR) using anthers at stage 9 to stage 10 to confirm that *TDR* and *GAMYB* mRNA are indeed m^6^A‐hypermethylated in *Osalkbh9‐1* compared to WT (Figure 6b). To validate *TDR* and *GAMYB* mRNA as direct targets of OsALKBH9, we performed RNA immunoprecipitation followed by RT‐qPCR (RIP‐RT‐qPCR) with an anti‐FLAG antibody in the anthers from *Osalkbh9‐1 OsALKBH9pro*::*OsALKBH9‐FLAG*. The results confirmed direct binding of OsALKBH9 to *TDR* and *GAMYB* mRNAs (Figure 6c). Note that we have found the higher expressed *TDR* and *GAMYB* mRNAs in *Osalkbh9‐1* (Figure 4c). As the mRNA m^6^A modification has been shown to promote mRNA stabilization in Arabidopsis and human, we assessed the mRNA stability of the *TDR* and *GAMYB* transcripts using actinomycin D in anthers. Our results showed that *TDR* and *GAMYB* transcripts are more stable in *Osalkbh9‐1* than in WT (Figure 6d), confirming that m^6^A modification mediates mRNA stabilization in rice. Collectively, our results demonstrate that disruption of OsALKBH9 increases m^6^A levels in *TDR* and *GAMYB* transcripts, which promotes mRNA stabilization and activates downstream gene expression of tapetum development, sporopollenin synthesis and transport, thereby leading to excessive accumulation of pollen exine (Figure 6e).

## Discussion

As the most abundant internal chemical modification found in eukaryotic mRNA, m^6^A modification plays vital roles in plants, including development, biotic and abiotic stress responses, as well as crop trait improvement (Tang *et al*., 2023). This reversible RNA modification can be removed by m^6^A demethylase. In mammals, two identified mRNA m^6^A demethylases are fat mass and obesity‐associated protein (FTO) (Jia *et al*., 2011) and alkylated DNA repair protein AlkB homologue 5 (ALKBH5) (Zheng *et al*., 2013). Arabidopsis has five homologues (ALKBH9A, ALKBH9B, ALKBH9C, ALKBH10A and ALKBH10B) of mammalian ALKBH5, but rice only has two homologues (OsALKBH9 and OsALKBH10) (Figure S1), suggesting low redundancy of m^6^A demethylases. To deeply explore and understand the roles of m^6^A demethylases in plants, it is necessary to study the functions of demethylases in rice, which serves as a model for monocotyledonous plants.

In this work, we identified OsALKBH9 as an mRNA m^6^A demethylase that plays a vital role in regulating male fertility in rice. Knockout of *OsALKBH9* resulted in delayed tapetal cell degradation and abnormal accumulation of pollen exine, ultimately leading to pollen abortion. The tapetum, which is the innermost cell layer of the anther, is essential for the development and maturation of microspores. During pollen development, the tapetum undergoes PCD. Early or delayed PCD of the tapetum causes abnormal microspore development. In the *Osalkbh9‐1* mutant, tapetum formation was normal, but its degradation was inhibited. The initiation time of tapetal PCD in the *Osalkbh9‐1* mutant was similar to that of the WT, starting from stage 8a. However, as measured by the TUNEL assays (Figure 2c), the tapetum degradation of *Osalkbh9‐1* was weaker than that of the WT, causing delayed tapetum degradation. In rice, tapetal PCD delayed mutants can be classified into two groups: tapetum expanded mutants, including *udt1* (Jung *et al*., 2005), *tdr* (Li *et al*., 2006), *gamyb* (Aya *et al*., 2009) and *tip2* (Fu *et al*., 2014), which exhibit resistance to tapetum degradation and little or no exine accumulation; and tapetum persistent mutants, including *ptc1* (Li *et al*., 2011), *eat1* (Niu *et al*., 2013), *ptc2* (Uzair *et al*., 2020) and *post* (Che *et al*., 2021), which have normal tapetum cell size and metabolic activity and these genes are highly expressed at post‐meiotic stages. While both *ptc1* and *ptc2* mutants accumulate less exine, *eat1* exhibited much thicker exine than that of WT. The *Osalkbh9‐1* male sterile mutant delayed degradation of tapetal cells and persistence at late stages, indicating that it is a tapetum‐persistent mutant. Interestingly, unlike most tapetal PCD delayed mutants, *Osalkbh9‐1* accumulated more exine on the pollen surface than WT (Figure 2b), suggesting excessive activation of pollen exine synthesis and/or transport pathways in *Osalkbh9‐1*. Indeed, we found OsALKBH9 can directly remove m^6^A in *TDR* and *GAMYB* mRNA, which reduces their stability and promotes accurate expression of downstream genes. In the mutant, m^6^A levels in *TDR* and *GAMYB* transcripts increased, leading to mRNA stabilization and activation of *OsMS188*, *PTC1*, *ABCG15*, *DPW*, *PKS1*, *CYP703A3* and *CYP704B2* expression, ultimately causing excessive accumulation of pollen exine. A recent study in Maize is similar to our results. In *Msms1* mutant, the tapetal PCD is delayed and the exine is aberrantly thickened (Hou *et al*., 2023). By map‐based clone, they identified a male‐sterility mutant gene, ZmMS1, encoding a tapetum‐specific lateral organ boundaries domain transcription factor ZmLBD30. Further study found that several tapetal development and pollen exine formation genes are upregulated in the mutant, causing an aberrantly thickened exine. Furthermore, ZmMS1/LBD30 serves as a repressor to shut down ZmbHLH51‐ZmMYB84‐ZmMS7 cascade to ensure timely tapetal degeneration and the proper level of exine (Hou *et al*., 2023).

In mammalians, both ALKBH5 and FTO are nuclear localization protein (Jia *et al*., 2011; Zheng *et al*., 2013), however, several mRNA m^6^A demethylase in plants, such as AtALKBH9B (Martinez‐Perez *et al*., 2017), SlALKBH2 (Zhou *et al*., 2019) and OsALKBH9, are localized in the cytoplasm, suggesting different functions of demethylases may exist in plants. AtALKBH9B was found to be localized with SGS3, a marker of stress granule (SG) and cytoplasmic siRNA body, implying a functional association with siRNA‐mediated RNA decay, RNA storage or mRNA translation. A recent work found that AtALKBH9B could demethylase the m^6^A of a heat‐activated long terminal repeat retrotransposon RNA (*Onsen*) in SG, releasing *Onsen* RNA out of SG (Fan *et al*., 2023). In this work, we also found OsALKBH9 co‐localized with SG, suggesting OsALKBH9 may involve SG related functions.

The precise regulation of mRNA m^6^A modification is crucial for rice fertility. In addition to *OsALKBH9*, other studies have also highlighted the significant roles of mRNA m^6^A modifications in rice male fertility. Knocking out of *OsFIP37*, a core component of m^6^A methyltransferase, leads to abnormal meiosis and early degeneration of microspores during the vacuolated pollen stage (Cheng *et al*., 2022; Zhang *et al*., 2019). Further studies demonstrated that OsFIP37 is recruited by OsFAP1 to mediate m^6^A modification on an auxin biosynthesis gene *OsYUCCA3* during microsporogenesis (Cheng *et al*., 2022). Deficiency of ENHANCED DOWNY MILDEW 2‐LIKE (OsEDM2L), an *N* ^6^‐adenine methyltransferase‐like domain‐containing protein essential for proper mRNA m^6^A modification in anthers, results in delayed tapetal PCD and defective pollen development in rice (Ma *et al*., 2021).

## Methods

### Plant materials and growth conditions

The rice mutants *Osalkbh9‐1* and *Osalkbh9‐2* were created by a clustered regularly interspaced short palindromic repeats (CRISPR)/Cas9 genome editing method in the background of *Japonica* rice ‘Nipponbare’. *Osalkbh9‐1* was obtained from Baige Gene Technology (Jiangsu, China) by CRISPR/Cas9 technology with target ‘ACGCGATGGCGCTCGTGCAGGGG’. *Osalkbh9‐2* was obtained from BIORUN (Wuhan, China) by CRISPR/Cas9 technology with target ‘GTACGTATACGGAACCCCAAAGG’. The *Osalkbh10* mutants were obtained from Baige Gene Technology (Jiangsu, China) by CRISPR/Cas9 technology with target ‘CCCTATCTGCTCTGATCACGAGG’ and ‘CACAACTTCAGAGCTCATGAAGG’. The CRISPR‐Cas9 T‐DNA free *Osalkbh9‐1* plants were obtained by screening plants without hygromycin bands through PCR, the primers are listed in Table S6. All plant materials were grown in the fields located in Beijing, China, and maintained with routine management practices.

### Phenotypic characterization and cytological observation

Pollen grains were stained by 1% (w/v) I_2_‐KI and observed by a lab microscope. The anther semi‐section, TEM analysis, TUNEL assay and the RNA *in situ* hybridization were performed as described previously (Bai *et al*., 2019). GUS activity was measured by staining spikelets at different stages of T_1_ transgenic lines as described previously (Bai *et al*., 2019).

### Plasmid construction and plant transformation

To generate *OsALKBH9pro*::*OsALKBH9CDS‐FLAG* construct. Firstly, we cloned the coding sequence (CDS) of *OsALKBH9* into vector pCAMBIA1300‐35S‐3 × Flag by *Kpn*I and *Sal*I, and we obtained pCAMBIA1300‐35S‐OsALKBH9‐3 × Flag construct. Then the 35S promotor of pCAMBIA1300‐35S‐OsALKBH9‐3 × Flag was replaced by a 2068‐bp fragment upstream ATG start codon of *OsALKBH9* by *Hind*III and *Kpn*I. We created the catalytically inactive mutant, *OsALKBH9pro*::*OsALKBH9CDSmut‐FLAG*, by introducing the OsALKBH9 H324A/D326A mutation using the Mut Express II Fast Mutagenesis Kit V2 (Vazyme). Both constructs were transformed into *Osalkbh9‐1*/*Osalkbh9‐1* calli, respectively. To construct *OsALKBH9pro*::*GUS*, we cloned the promoter fragments into pCAMBIA1300‐GUS‐3U (Tang *et al*., 2020) by *Xba*I and *Bam*HI, and transformed them into Nipponbare. All primers used for vector construction were listed in Table S6.

### LC‐MS/MS for m^6^A quantification

The m^6^A quantification by LC–MS/MS was performed as described previously (Wang *et al*., 2022). Poly A^+^ RNA (200 ng) was digested with 1 U Nuclease P_1_ in 40 μL of buffer containing 10% 0.1 M ammonium acetate NH_4_AC (pH 5.3) at 42 °C for 4 h, followed by the addition of 2 U Shrimp Alkaline Phosphatase (NEB, USA) and 10× CutSmart buffer (NEB). The mixture was incubated at 37 °C for 3 h, and the resulting aqueous phase was injected into an LC–MS/MS system. Nucleosides were separated using a UPLC pump (Shimadzu, Japan) with a ZORBAX SB‐Aq column (Agilent, USA) and analysed by MS/MS using a Triple QuadTM 5500 (AB SCIEX, USA) mass spectrometer running in positive ion mode and the multiple reaction‐monitoring (MRM) feature.

### Protein purification and *in vitro* demethylation assays

The coding sequence of *OsALKBH9* was amplified and cloned into pET28a^+^. The resulting construct, Pet28a^+^‐OsALKBH9 was transformed into *E. coli* strain BL‐21 Gold competent cells, and induced by 0.5 mM IPTG at 18 °C overnight. The protein purification process followed the procedures outlined in the Ni‐NTA Spin Kit handbook (QIAGEN).

Demethylation activity assays were performed with limited modifications as previously reported (Zheng *et al*., 2013). Briefly, reaction mixtures contained the following components: 0.5 μL oligo RNA with m^6^A (AUUGUCA(m^6^A) CAGCAGC) or 500 ng full‐length rice mRNA, OsALKBH9, KCl (100 μM), MgCl2 (2 mM), RNasin (0.2 U μL −1, Thermofisher), l‐ascorbic acid (200 μM), a‐ketoglutarate (300 μM), (NH4)_2_Fe (SO4)_2_ (150 μM) and 50 mM of HEPES buffer (PH 7.0). The reaction was incubated at room temperature overnight, the quenched by adding 5 mM EDTA followed by heating at 95 °C for 10 min, and analysed by LC–MS/MS.

### RNA extraction and qRT‐PCR

Total RNA was extracted from anthers at stage 7 to stage 8 and stage 9 to stage 10 using Quick‐RNA™ Plant Miniprep kit (Zymo research). One microgram of total RNA was used for reverse transcription by PrimeScript RT reagent kit with gDNA Eraser (Takara). qRT‐PCR was performed using SYBR Green Master Mix (YEASEN) on a ViiA 7 Dx (Applied Biosystems). The 2^−ΔΔ*CT*^ method was used to calculate the gene expression levels. All primers were designed by multiPrime at http://www.multiprime.cn (Xia *et al*), and listed in (Table S6).

### RNA seq and data analysis

Ribosomal RNA was depleted from total RNA samples extracted from stage 7 to stage 8 and stage 9 to stage 10 using riboPOOL (siTOOLs Biotech). The remaining rRNA‐depleted RNA was used to generate libraries with a NEBNext Ultra II RNA Library Prep Kit, followed by paired‐end sequencing on an Illumina HiSeq X Ten instrument with 150 bp per read (Genewiz). Two biological replicates were performed for each sample.

The obtained sequencing reads were preprocessed using Cutadapt (v1.18) to remove adapter sequences and low‐quality bases. High‐quality reads were subsequently aligned to the *Oryza_sativa* IRGSP‐1.0 reference genome using HISAT2 (v2.1.0), while PCR duplicates were removed with the Picard Toolkit. Differentially expressed genes were identified with the R package DESeq2, and Gene Ontology (GO) enrichment analysis was conducted using the plant‐regulomics (http://bioinfo.sibs.ac.cn/plant‐regulomics/).

### m^6^A‐seq and data analysis

Poly A^+^ RNA was enriched from total RNA using Oligo(dT)25 Dynabeads (Thermo Fisher Scientific). Subsequently, 500 ng Poly A^+^ RNA was fragmented into 100 nt fragments using a Magnesium RNA fragmentation module (NEB). Immunoprecipitation (IP) of m^6^A was conducted with an EpiMark *N* ^6^‐Methyladenosine enrichment kit (NEB), and the RNA eluted from the m^6^A‐IP and input RNA were used to prepare libraries with the NEBNext Ultra II RNA Library Prep Kit. Paired‐end sequencing was performed on an Illumina HiSeq X Ten platform with 150 bp per read (Genewiz).

Cutadapt (v1.18) was used to preprocess the raw sequencing reads and remove adapter sequences and low‐quality bases. The resulting high‐quality reads were aligned to the *Oryza_sativa* IRGSP‐1.0 reference genome using HISAT2 (v2.1.0). The resulting mapping reads were utilized to identify m^6^A peaks with the R package EXOMEPEAK, and MeTDiff was employed to detect differential m^6^A peaks based on criteria of fold change >2 and FDR < 0.05. Peak annotation was performed using Bedtools and custom python scripts available at https://github.com/joybio/m6A‐seq/tree/main/feature_annotation/. Gene Ontology (GO) functional annotations were conducted using the plant‐regulomics database (http://bioinfo.sibs.ac.cn/plant‐regulomics/), while motifs were identified with HOMER.

### m^6^A‐IP‐qPCR

The procedure utilized in this study was based on the previously described m^6^A‐seq method (Dominissini *et al*., 2013). Briefly, 50 μg total RNA was fragmented to 100–150 nt using RNA Fragmentation Reagents (Invitrogen), followed by ethanol precipitation. Subsequently, 10% of the eluted RNA was saved as input sample, while the remaining RNA was incubated with 5 μg m^6^A antibody (#202203; Synaptic Systems) in 500 μL IP buffer 750 mM NaCl, 0.5% Igepal CA‐630, 50 mM Tris–HCl (pH 7.4), 200 U RiboLock RNase Inhibitor (Thermo Fisher) at 4 °C for 2 h. The m^6^A‐containing fragments were pulled down with Dynabeads Protein A and eluted with RLT buffer, followed by ethanol precipitation. Both input and IP samples were analysed by qRT‐PCR using primers listed in Table S6. The relative enrichment of m^6^A in each sample was calculated by normalizing the value of the amplification cycle (Cq) of the m^6^A‐IP portion to the Cq of the corresponding input portion.

### RIP‐qPCR

The RNA immunoprecipitation was carried out according to previously described method (Koster and Staiger, 2014). Briefly, spikelets (anthers at stage 7 to stage 12) of *Osalkbh9‐1* and NIP were harvested and fixed in 1% formaldehyde under vacuum for 15 min. Subsequently, they were terminated with 150 mM glycine for 10 min. Two grams of fixed material was homogenized in 2 mL of lysis buffer (50 mM HEPES, pH 7.5, 150 mM KCl, 2 mM EDTA, 0.5% Igepal CA‐630, 0.5 mM DTT, 1× Roche Protease inhibitor cocktail and 40 U/mL Ribolock RNase inhibitor). The extract was centrifuged at 13,000 rpm for 10 min at 4 °C. A part of the lysate was taken as input samples, while the rest was divided into two equal volumes and subsequently immunoprecipitated with either anti‐Flag M2 magnetic beads (Sigma‐Aldrich, USA) or normal rabbit IgG (Cell Signaling Technology, USA) bounded to Dynabeads Protein A. After washing and ethanol precipitation, the recovered RNA fractions were used for qRT‐PCR. LOC_Os02g37950, which was devoid of an m^6^A peak from m^6^A profiling data, was employed as the internal control.

### mRNA stability assay

The procedure was based on the previously described method (Duan *et al*., 2017). Briefly, spikelets (anthers at stage 7 to stage 12) of *Osalkbh9‐1* and NIP were harvested and transferred to 1/2 MS liquid medium. A final concentration of 100 μM actinomycin D was added to the medium, followed by infiltration for 1 h. The spikelets were then collected and considered as time 0 controls, while subsequent samples were collected every 2 h. qRT‐PCR was used to determine the remaining mRNA levels, with 18S rRNA serving as a reference.

## Author contributions

G.J., J.W., S.Z. and J.T. conceived the project; J.T. and D.L. performed the experiments with the help of S.C., X.W., X.H., S.Z. and Z.C.; J.Y. analysed the sequencing data; J.T., D.L., J.Y., G.J., J.W. and S.Z. designed the experiments, interpreted the results, and wrote the manuscript. All authors read and approved the final manuscript.

## Conflict of interest

The authors declare no competing interests.

## Supporting information


**Figure S1** Sequence alignment of the AlkB domain proteins.
**Figure S2** CRISPR/Cas9‐mediated target mutagenesis of *OsALKBH10*.
**Figure S3** Pollen grains of genetically complementary plants and demethylase inactive complementary plants.
**Figure S4** Subcellular localization of OsALKBH9‐eGFP in *N. benthamiana* leaves epidermal cells.
**Figure S5** Correlation of RNA‐seq data.
**Figure S6** PCA analysis of RNA‐seq data.
**Figure S7** Hierarchical clustering and Gene Ontology analysis of RNA‐seq data.
**Figure S8** Joint analysis of m^6^A‐seq and RNA‐seq.
**Table S1** The genetic segregation ratios of genotypes and phenotypes from heterozygotes.
**Table S2** List of RNA‐seq data of genes corresponding to Figure 4C.

**Table S3** RNA‐seq of anthers at S7‐8.
**Table S4** RNA‐seq of anthers at S9‐10.
**Table S5** m^6^A hypermethylated peaks.
**Table S6** Primers used in this study.

## Data Availability

The raw sequencing data reported in this paper have been deposited in the Genome Sequence Archive in the National Genomics Data Center (NGDC), China National Center for Bioinformation/Beijing Institute of Genomics, Chinese Academy of Sciences (GSA: CRA010684) which is publicly accessible at https://ngdc.cncb.ac.cn/gsa.
