# Supplementary material for: OsALKBH9‐mediated m6A demethylation regulates tapetal PCD and pollen exine accumulation in rice
Source: Plant Biotechnol J. 2024 Apr 17;22(9):2410–23. doi: 10.1111/pbi.14354 (PMC11332222; doi:10.1111/pbi.14354)
Supplement: Supplementary file 1 — Figure S1 Sequence alignment of the AlkB domain proteins. Figure S2 CRISPR/Cas9‐mediated target mutagenesis of OsALKBH10. Figure S3 Pollen grains of genetically complementary plants and demethylase inactive complementary plants. Figure S4 Subcellular localization of OsALKBH9‐eGFP in N. benthamiana leaves epidermal cells. Figure S5 Correlation of RNA‐seq data. Figure S6 PCA analysis of RNA‐seq data. Figure S7 Hierarchical clustering and Gene Ontology analysis of RNA‐seq data. Figure S8 Joint analysis of m6A‐seq and RNA‐seq. Table S1 The genetic segregation ratios of genotypes and phenotypes from heterozygotes. Table S2 List of RNA‐seq data of genes corresponding to Figure 4C. [file PBI-22-2410-s002.doc]

**Supplemental information (SI)**

**
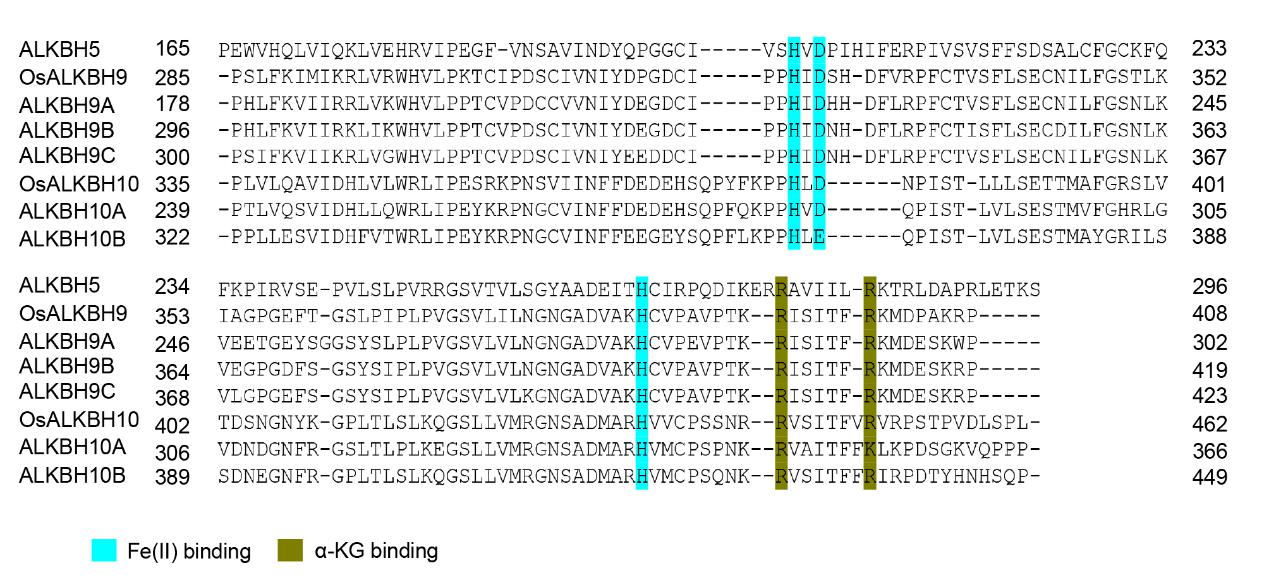
**

**Supplemental Figure 1. Sequence alignment of the AlkB domain proteins.**

Sequence alignment of the AlkB domain of human ALKBH5 with five Arabidopsis and two rice AlkB family proteins. Conserved residues are indicated by vertical lines. Iron

1. binding sites and α-KG binding sites are highlighted by blue and brown, respectively.

1


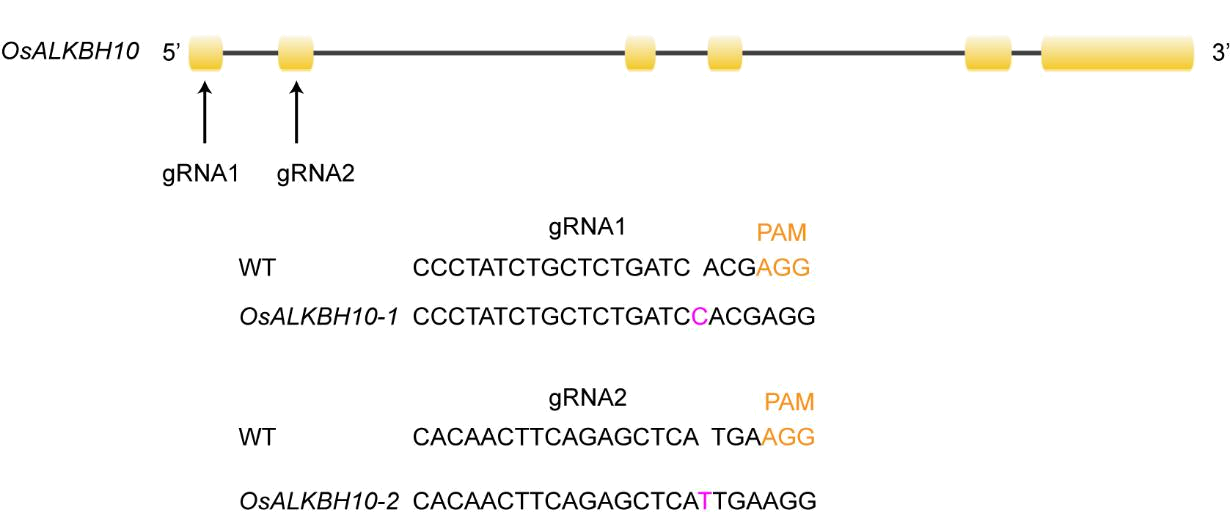


**Supplemental Figure 2. CRISPR/Cas9-mediated target mutagenesis of**

***OsALKBH10***. The upper panel shows the *OsALKBH10* genomic region and the two CRISPR/Cas9 targets sites indicated by arrows. Exons and other sequences are indicated by yellow boxes and black lines, respectively. The lower panel shows alignment of wild-type (WT), *Osalkbh10-1*, and *Osalkbh10-2* sequences containing the CRISPR/Cas9 target sites. *Osalkbh10-1* and *Osalkbh10-2* contain a 1-bp insertion of C (red) and a 1-bp insertion of T (red), respectively.

2


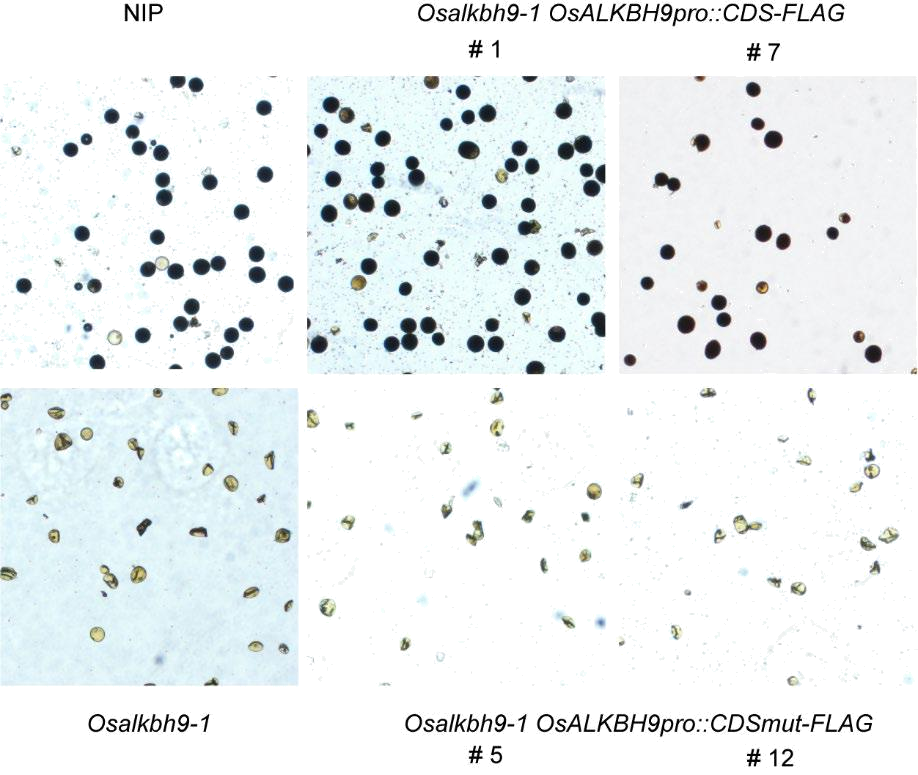


**Supplemental Figure 3. Pollen grains of genetically complementary plants and demethylase inactive complementary plants**, stained by I2/KI solution.

3


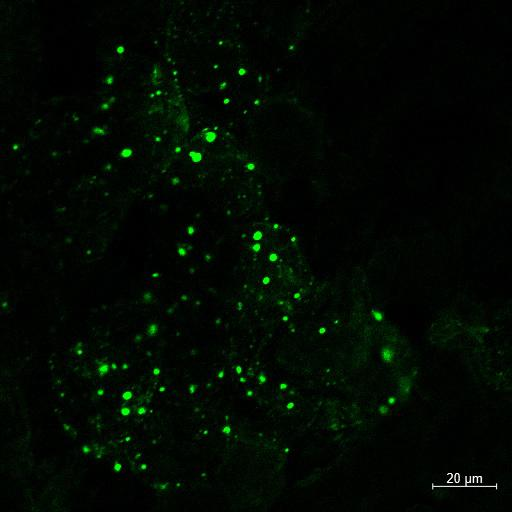


**Supplemental Figure 4. Subcellular localization of OsALKBH9-eGFP in *N.***

***benthamiana* leaves epidermal cells.**

4


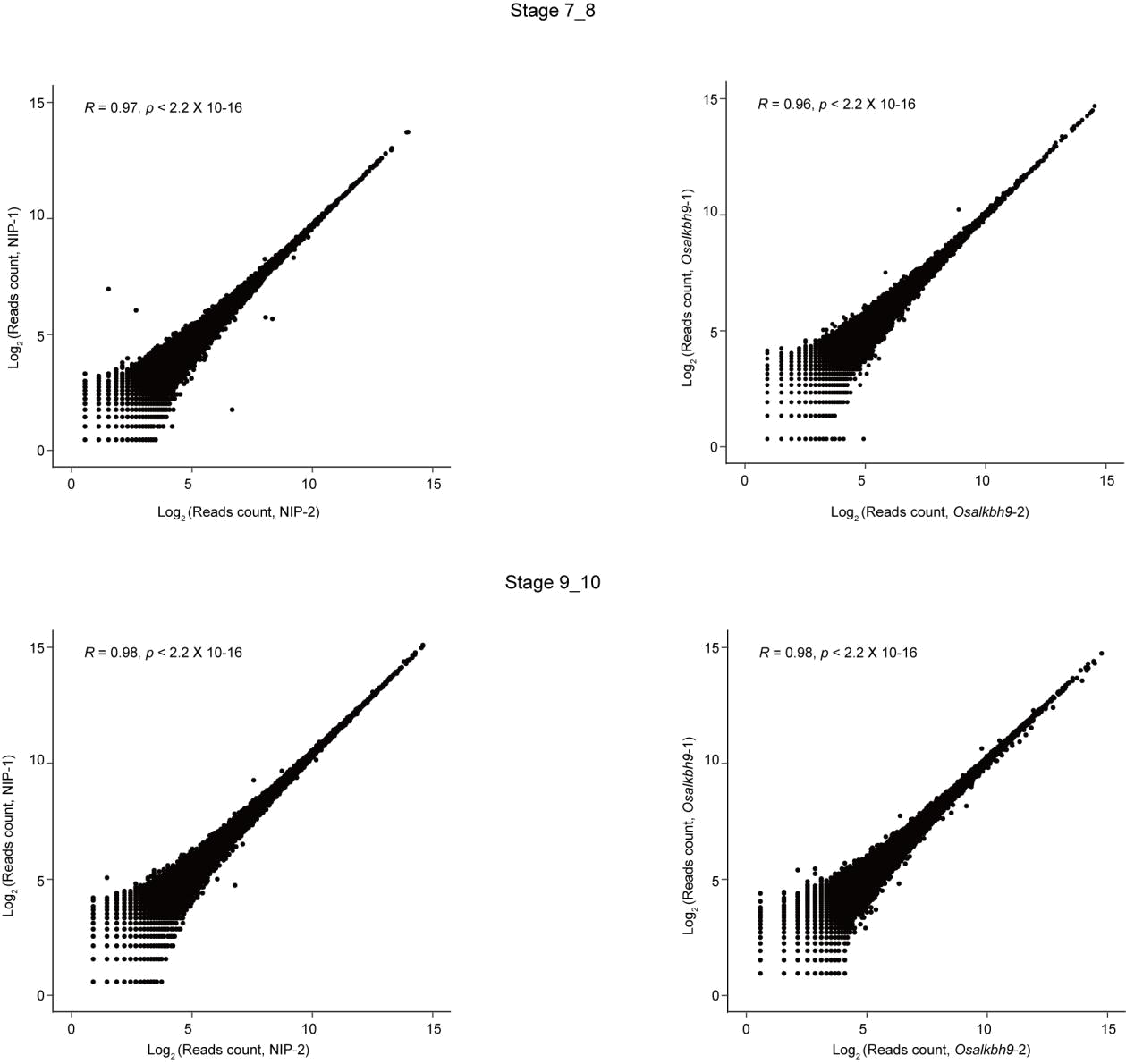


**Supplemental Figure 5**. **Correlation of RNA-seq data**.

Correlation of mRNA FPKM between two biological replicates in WT and *Osalkbh9-1* at Stage 7-8 and Stage 9-10.

5


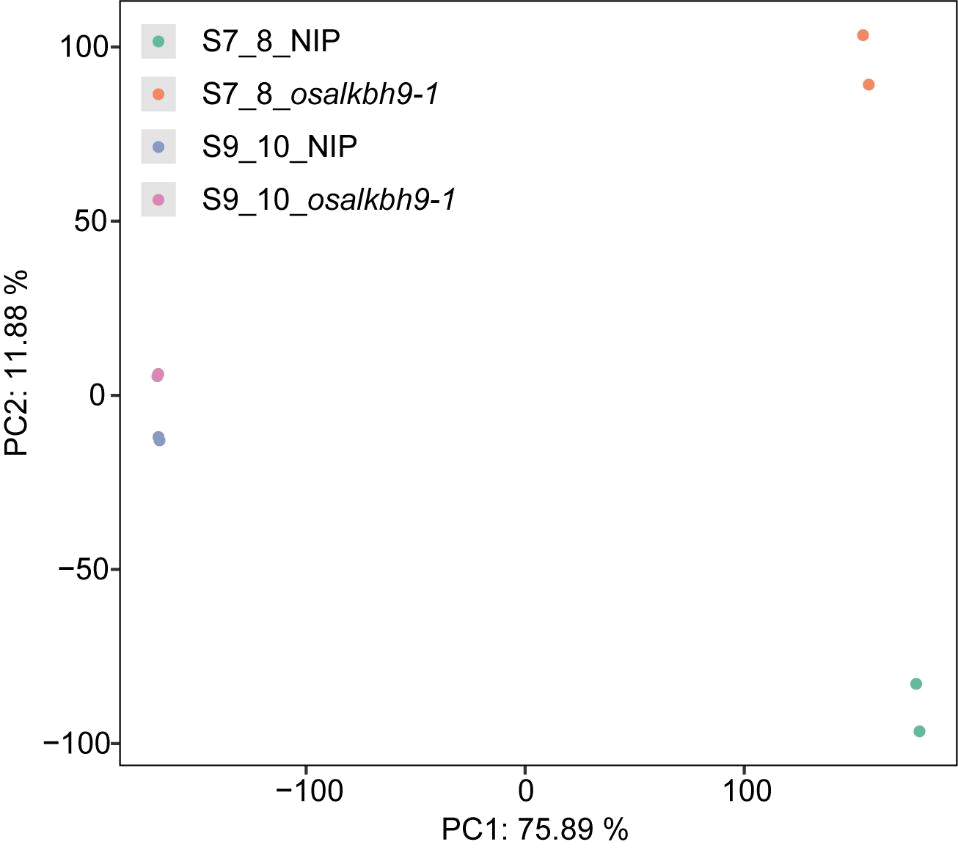


**Supplemental Figure 6**. **PCA analysis of RNA-seq data**. The principal component analysis (PCA) revealed that samples from distinct stages could be categorized into two subgroups by PC1 (supplementary figure), constituting 75.89% of the variance. Additionally, PC2 demonstrated the capability to distinguish between NIP and *Osalkbh9*-1 within each stage, contributing to 11.88% of the variance.

6


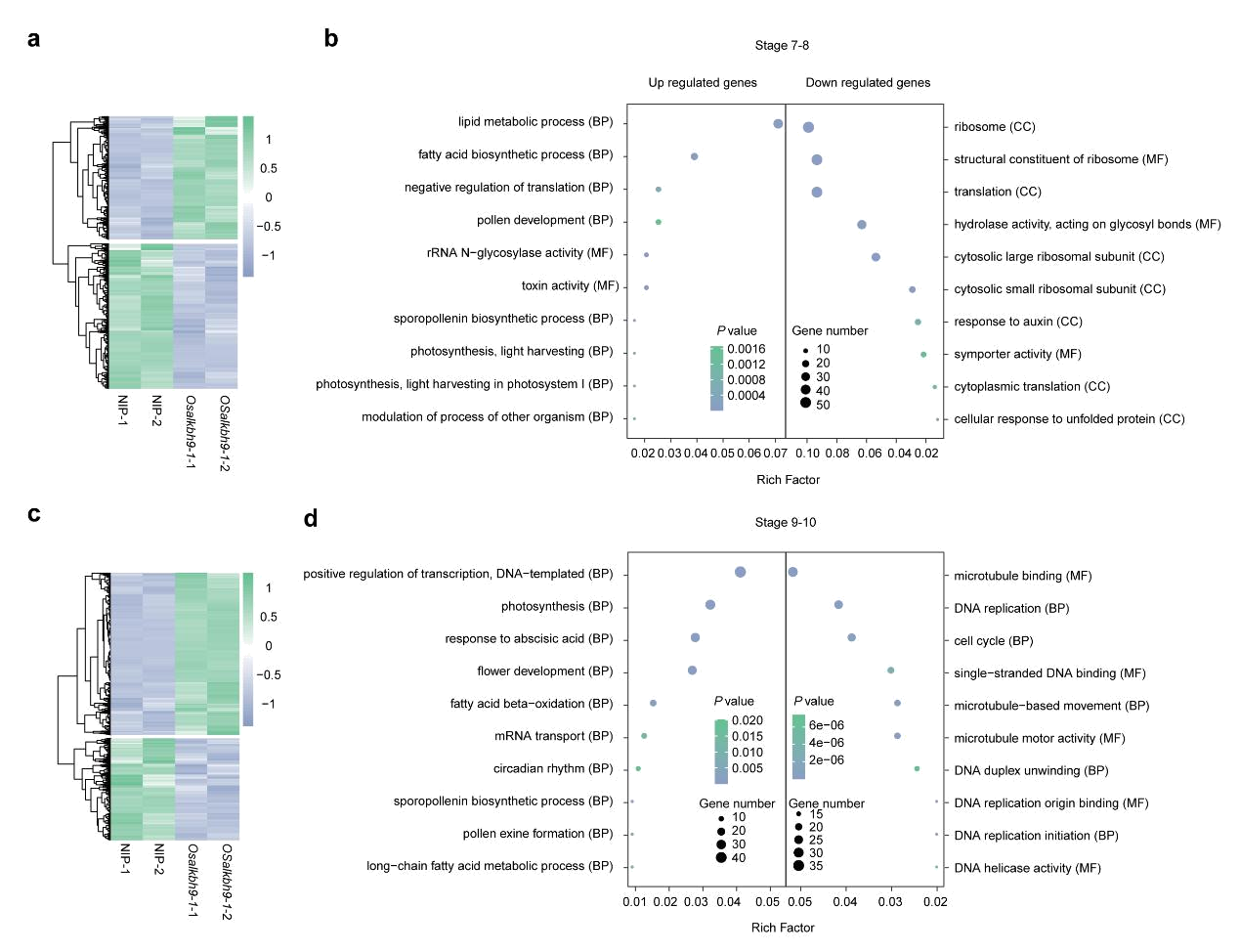


**Supplemental Figure 7**. **Hierarchical clustering and Gene Ontology analysis of RNA-seq data.** Hierarchical clustering (**a, c**) and Gene Ontology analysis (**b, d**) of differential expressed genes at Stage 7-8 and Stage 9-10.


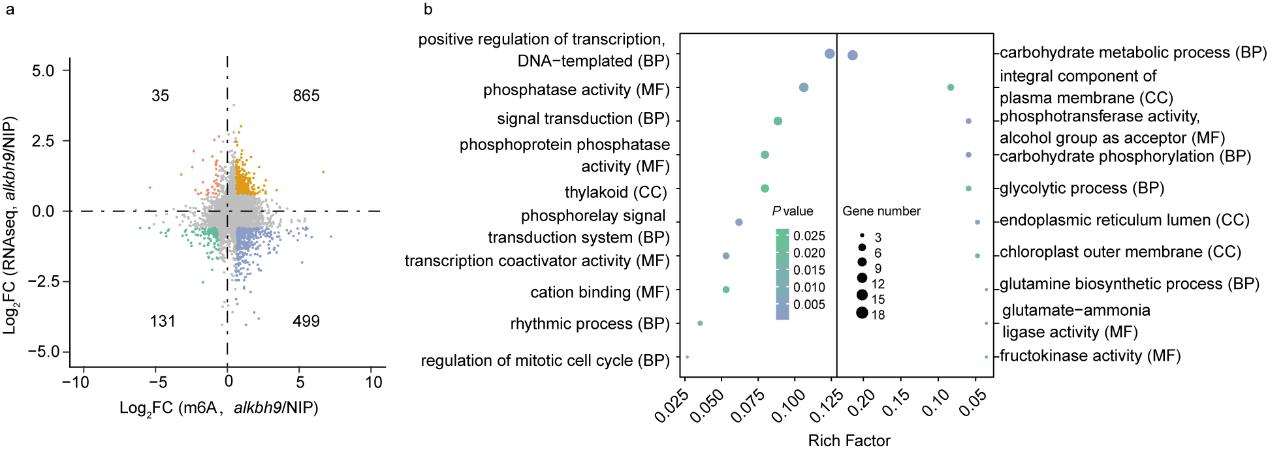


**Supplemental Figure 8**. **Joint analysis of m^6^A-seq and RNA-seq.**

1. Joint analysis of m^6^A-seq and RNA-seq in *O*s*alkbh9-1* and WT. DEGs (fold-change

≥ 1.5 and P < 0.05) with differentially methylated m^6^A peaks (fold-change ≥ 1.5 and

P < 0.05) are highlighted. **(b)** GO enrichment analysis of DEGs with differential m^6^A methylation in *alkbh9*.

7

**Supplemental Table 1. The genetic segregation ratios of genotypes and phenotypes from heterozygotes**

| Mutant | Wild | Heterozygote | Homozygote Segregation | χ2 |
| --- | --- | --- | --- | --- |
| materials | type |  | ratio |  |
|  |  |  |  |  |
| *Osalkbh9-1* | 45 | 91 | 43 | 0.09 |
| Grain fertility | Fertile | Fertile | 1:2:1 |  |
|  |  |  | Sterile |  |
|  | 38 | 80 | 42 |  |
| *Osalkbh9-2* |  |  |  | 0.13 |
| Grain fertility | Fertile | Fertile | 1:2:1 |  |
|  |  |  | Sterile |  |

Note: Chi-square test for the ratio of 3 (fertile): 1 (sterile), P = 0.05, χ^2^ = 3.8.

**Supplemental Table 2. List of RNA-seq data of genes corresponding to Figure 4C**

| MSU ID | Gene name | Log2FC(*Osalkbh9-1*/WT) | |
| --- | --- | --- | --- |
|  |  | Stage 7-8 | Stage 9-10 |
|  |  |  |  |
| LOC_Os01g68870 | *MSP1* | 0.233 | 0.037 |
| LOC_Os07g36460 | *UDT1* | -0.744 | 0.122 |
| LOC_Os03g18480 | *TDF1* | 1.42 | 6.23 |
| LOC_Os02g02820 | *TDR* | 0.687 | 0.887 |
| LOC_Os01g59660 | *GAMYB* | -0.203 | 1.646 |
| LOC_Os01g18870 | *TIP2* | 0.760 | -0.054 |
| LOC_Os03g50780 | *TIP3* | 0.982 | 0.622 |
| LOC_Os04g51070 | *EAT1* | 0.798 | -0.066 |
| LOC_Os04g39470 | *MS188* | 1.626 | 5.367 |
| LOC_Os09g27620 | *PTC1* | 0.827 | 3.264 |
| LOC_Os10g34360 | *PKS1* | 1.390 | 4.029 |
| LOC_Os07g22850 | *PKS2* | 2.018 | 5.246 |
| LOC_Os06g40550 | *ABCG15* | 1.422 | 4.001 |
| LOC_Os08g03682 | *CYP703A3* | 2.555 | 6.096 |
| LOC_Os03g07250 | *CYP704B2* | 0.973 | 2.950 |
|  |  | 8 |  |
